# Efficacy of the unified protocol for transdiagnostic cognitive-behavioral treatment for depressive and anxiety disorders: a randomized controlled trial

**DOI:** 10.1017/S0033291721005067

**Published:** 2023-05

**Authors:** Masaya Ito, Masaru Horikoshi, Noriko Kato, Yuki Oe, Hiroko Fujisato, Keiko Yamaguchi, Shun Nakajima, Mitsuhiro Miyamae, Ayaka Toyota, Yasuyuki Okumura, Yoshitake Takebayashi

**Affiliations:** 1 National Center for Cognitive Behavior Therapy and Research, National Center of Neurology and Psychiatry, Tokyo, Japan; 2 Department of Neuropsychiatry, Keio University School of Medicine, Tokyo, Japan; 3 Department of Neuropsychiatry, Kyorin University School of Medicine, Tokyo, Japan; 4 National Institute of Mental Health, National Center of Neurology and Psychiatry, Tokyo, Japan; 5 Department of Functional Brain Imaging, National Institutes for Quantum and Radiological Science and Technology, Chiba, Japan; 6 Initiative for Clinical Epidemiological Research, Tokyo, Japan; 7 Department of Health Risk Communication, School of Medicine, Fukushima Medical University, Fukushima, Japan

**Keywords:** Anxiety, cognitive behavioral therapy, depression, transdiagnostic, unified protocol

## Abstract

**Background:**

The efficacy of the unified protocol of the transdiagnostic treatment for emotional disorders (UP) has been poorly studied in patients with depressive disorders. This study aimed to examine the efficacy of UP for improving depressive symptoms in patients with depressive and/or anxiety-related disorders.

**Methods:**

This assessor-blinded, randomized, 20-week, parallel-group, superiority study compared the efficacy of the UP with treatment-as-usual (UP-TAU) *v.* wait-list with treatment-as-usual (WL-TAU). Patients diagnosed with depressive and/or anxiety disorders and with depressive symptoms participated. The primary outcome was depressive symptoms assessed by GRID-Hamilton depression rating scale (GRID-HAMD) at 21 weeks. The secondary outcomes included assessor-rated anxiety symptoms, severity and improvement of clinical global impression, responder and remission status, and loss of principal diagnosis.

**Results:**

In total, 104 patients participated and were subjected to intention-to-treat analysis [mean age = 37.4, s.d. = 11.5, 63 female (61%), 54 (51.9%) with a principal diagnosis of depressive disorders]. The mean GRID-HAMD scores in the UP-TAU and WL-TAU groups were 16.15 (s.d. = 4.90) and 17.06 (s.d. = 6.46) at baseline and 12.14 (s.d. = 5.47) and 17.34 (s.d. = 5.78) at 21 weeks, with a significant adjusted mean change difference of −3.99 (95% CI −6.10 to −1.87). Patients in the UP-TAU group showed significant superiority in anxiety and clinical global impressions. The improvement in the UP-TAU group was maintained in all outcomes at 43 weeks. No serious adverse events were observed in the UP-TAU group.

**Conclusions:**

The UP is an effective approach for patients with depressive and/or anxiety disorders.

## Introduction

Depressive and anxiety disorders represent one of the greatest burdens among human diseases worldwide (World Health Organization, 2017). These emotionally difficult conditions often manifest as comorbidities (Kessler et al., 2005; Lamers et al., 2011). Advances in understanding the genetic, biological, neurological, and psychological aspects of psychopathologies have indicated that transdiagnostic or higher-order features are shared across a variety of mental disorders (Caspi & Moffitt, 2018; Grisanzio et al., 2018; Insel et al., 2010; Krueger et al., 2018; McGorry, Hartmann, Spooner, & Nelson, 2018; Sloan et al., 2017; Watson, 2005). In parallel with the transdiagnostic understanding of psychopathology across ‘emotional disorders’ [i.e. major depressive disorder (MDD), generalized anxiety disorder (GAD), panic disorder (PD), social anxiety disorder (SAD), other anxiety-related disorders, post-traumatic stress disorder (PTSD), and obsessive-compulsive disorder (OCD)], accumulating evidence indicates that the transdiagnostic approach for treating these disorders is safe, feasible, and efficient (Newby, McKinnon, Kuyken, Gilbody, & Dalgleish, 2015). However, non-negligible methodological issues have been observed in the clinical trials of transdiagnostic psychotherapies (Fusar-Poli et al., 2019).

The unified protocol of transdiagnostic treatment for emotional disorders (UP) is an emotion-focused treatment consisting of the intervention modules derived from cognitive behavioral therapy (CBT) (Barlow et al., 2011). These treatment modules target the patients' negative emotionality and aversive reactions to emotions when they occur. A large clinical trial (*n* = 225) showed equivalent efficacy of the individual format of UP compared to established disorder-specific CBT and superior efficacy compared to wait-list conditions in patients with PD, SAD, GAD, and OCD (Barlow et al., 2017). The evidence for the feasibility of UP has accumulated for multiple formats (e.g. individual, group, and online) among diverse clinical populations (e.g. patients with anxiety, obsessive-compulsive, trauma-related, depressive, bipolar, borderline personality, and eating disorders) in various settings (e.g. public health services, ambulatory care, and inpatient care) and among different countries (e.g. the US, Japan, Brazil, Spain, Iran, Denmark, and Romania) (Barlow & Farchione, 2017; Cassiello-Robbins, Southward, Tirpak, & Sauer-Zavala, 2020; Sakiris & Berle, 2019).

Although UP is hypothesized to be transdiagnostically effective for a range of mental disorders associated with deficits in emotion regulation, evidence on the efficacy of UP for patients with a principal diagnosis of depressive disorder is limited (Sauer-Zavala et al., 2020). Only two randomized controlled trials have reported the efficacy of individual format of UP for patients with a principal diagnosis of depressive disorders (total *n* of UP sample = 25) (Bameshgi, Kimiaei, & Mashhadi, 2019; Marnoch, 2014), whereas seven randomized controlled trials have reported the efficacy of individual format of UP for patients with a principal diagnosis of anxiety-related disorders (total *n* of UP sample = 251) (Cassiello-Robbins et al., 2020; Sakiris & Berle, 2019). These two depression trials were conducted with nonblinded assessment and no fidelity rating of the intervention (Bameshgi et al., 2019; Marnoch, 2014). Moreover, these two trials represented limited clinical populations (i.e. depressed women due to marital problems and people aged ⩾60 years). To date, no clinical trial has been conducted with a rigorous design (e.g. blinded independent evaluator assessment, allocation concealment, large sample size, and fidelity assessment) including both patients with a principal diagnosis of depressive disorder and those with a principal diagnosis of anxiety disorders. The randomized controlled trial of a group format of UP (*n* = 291) showed promising efficacy on well-being for patients with a principal diagnosis of MDD, SAD, and PD (Reinholt et al., 2021). Along with the transdiagnostic theorization of UP, we hypothesized that the individual format of UP is effective for patients with either a principal diagnosis of depressive or anxiety disorders.

From a global mental health perspective, it is important to test the efficacy of transdiagnostic psychotherapy outside of Western countries. Efficacy study into psychosocial interventions has predominantly been limited to North America, Western Europe, and Australia (Patel et al., 2007; Saxena, Thornicroft, Knapp, & Whiteford, 2007). The lack of evidence from other regions may be among the reasons for the scarcity of psychotherapy dissemination in other parts of the world. Transdiagnostic psychotherapies, such as UP, have several potential advantages over disorder-specific treatment, especially for those countries with limited recourses and sparse efficacy data (Martin, Murray, Darnell, & Dorsey, 2018; Murray, Metz, & Callaway, 2019). Potential strengths of the transdiagnostic approach include applicability to diverse disorders, effective and efficient strategies for dealing with comorbidities, simplification of treatment models for multiple emotional disorders, ease of learning and training for novice therapists, reduction of confusion around what evidence-based treatment to choose, capitalization on the similarities across diagnoses and treatments within existing disorder-specific treatments, and cost reduction in comparison to training practitioners in multiple treatment models (McHugh & Barlow, 2010; Murray et al., 2019). Owing to these potential benefits, the transdiagnostic approach is expected to improve mental healthcare in low, middle, and high-income countries (Martin et al., 2018; Murray et al., 2019).

Hence, we aimed to test whether the addition of UP to treatment-as-usual (UP-TAU) would be more efficacious than wait-list with treatment-as-usual (WL-TAU) for reducing depressive symptoms, as assessed by the GRID-Hamilton depression rating scale (GRID-HAMD) (Williams et al., 2008), in Japanese patients with a principal diagnosis of depressive or anxiety disorders.

## Materials and methods

### Design

This study was designed as a single-centre, assessor-blinded, randomized, parallel-group superiority trial with a target sample of 104 patients with depressive and/or anxiety disorders with a primary time point of 21 weeks and a secondary follow-up period of 43 weeks. The participants were recruited from December 2013 to March 2018. The detailed study protocol, including the rationale of the comparison group, a detailed description of study settings, and the procedures, has been previously published (Ito et al., 2016). This trial was originally registered at ClinicalTrials.gov (NCT02003261) and later at the UMIN clinical trial registry (UMIN000030708) to comply with the change in the Japanese Ethical Guideline.

### Participants

The inclusion criteria were as follows: (1) a DSM-IV diagnosis of MDD, dysthymia, depressive disorder not otherwise specified, PD with agoraphobia, PD without agoraphobia, agoraphobia without history of PD, SAD, OCD, PTSD, GAD, or anxiety disorder not otherwise specified, as assessed by a Structured Clinical Interview for DSM-IV-TR Axis I Disorders (SCID-IV-TR) (First, Spitzer, Gibbon, & Williams, 1997); (2) mild or severe depressive symptoms (GRID-HAMD ⩾8); (3) aged 20–65 years old; and (4) provision of full informed consent to participate in the study. The exclusion criteria were as follows: (1) alcohol or substance abuse disorder in 6 months prior to the baseline; (2) current manic episode, schizophrenia, or other psychotic disorder; (3) serious suicidal ideation; (4) life-threatening, severe, or unstable physical disorders or major cognitive deficits; (5) evidence of an inability to participate in half or more of the intervention phase; (6) structured psychotherapy; and (7) other relevant reasons. We used the SCID-IV-TR to assess diagnostic status because the Japanese version of the DSM-5 and its structured instrument (e.g. SCID-5) had not been published. We included structured psychotherapy (i.e. psychotherapy following a specific pre-determined protocol, such as CBT) among the exclusion criteria to exclude possible effects due to other treatments.

Six independent evaluators, who were blinded to the allocation, conducted the SCID-IV-TR, GRID-HAMD, and other rating scales. All evaluated assessments were recorded, and 20% of the completed assessments were randomly selected to calculate the intraclass correlation coefficient using a two-way random-effects model for absolute agreement. Independent evaluators were unaware as to which interview sessions were to be re-evaluated for inter-rater consistency.

### Randomization and blinding

We employed central randomization using the Allocation and Registration Control System computer software, which was developed, set up, and managed by an independent institution (i.e. Keio University). A random sequence was generated using minimization, with a ratio of 1:1 to balance stratified factors (i.e. the principal diagnosis of depressive *v.* anxiety disorder). Allocation was implemented by the primary investigator (MI) or research coordinator via the internet using a laptop in front of the eligible participant. The independent evaluators responded to the modified version of the Independent Evaluator Knowledge of Treatment scale at mid- (10 weeks), post- (21 weeks), and follow-up assessment (43 weeks).

### Intervention

#### UP

UP is an emotion-focused CBT intervention that targets core mechanisms shared across depressive and anxiety disorders (Barlow et al., 2011). Standard administration comprises 12–18 weeks of 50–60 min weekly sessions. Participants learn cognitive behavioral skills to regulate their emotions by practicing the core components of UP, such as monitoring their emotional experience, mindful emotional awareness, cognitive flexibility, countering emotional behaviors, and interoceptive and emotional exposures. Four clinical psychologists conducted the individual format of UP (two females and two males, all with 4–6 years of clinical practice after certification). Treatment adherence was monitored by weekly group supervision and assessed using the Treatment Adherence Scale for UP (Barlow et al., 2017). To assess treatment adherence and fidelity, we selected 25% of the total expected sessions. This random sampling was blinded to the therapists. The overall adherence was calculated as the proportion of non-deviation from the treatment procedure in the assessed session. The treatment fidelity was assessed by the overall session rating, which ranged from poor to excellent (0–5). The intervention period was set at 20 weeks to allow participants to skip sessions in case of unforeseen circumstances (e.g. catching a cold) and to allow them to practice the UP skills independently with longer intervals between sessions later in the treatment period.

#### TAU

The Japanese Society of Mood Disorders has published treatment guidelines for MDD that recommend pharmacotherapy for moderate or severe MDD patients and pharmacotherapy and psychotherapy for mild MDD patients (Japan Society for Mood Disorders Treatment, 2013). However, there are no treatment guidelines for anxiety disorders specific to a Japanese clinical setting. In this clinical trial, we defined TAU as any pharmacological or psychological intervention and clinical management except structured psychotherapy and electroconvulsive therapy. For ethical reasons and to maintain clinical relevance specific to the Japanese clinical setting, all participants received TAU without any restriction on routine psychiatric treatment, including changes to the dose and types of psychotropic medicines. The primary psychiatrists who provided TAU did not blinded to the treatment allocation.

### Measures

#### Primary outcome

The primary outcome was depressive symptoms assessed by the 17-item version of GRID-HAMD at 21 weeks. We used GRID-HAMD because it assesses not only depressive symptoms (i.e. depressed mood, guilt, suicide, insomnia, difficulties in work and activities, psychomotor retardation or agitation, loss of appetite, sexual interest, weight, or insight) but also anxiety-related symptoms (i.e. psychic and somatic anxiety, general somatic symptoms, and hypochondriasis), which reflect common emotional symptoms across depressive and anxiety disorders (range: 0–52). The GRID-HAMD has been reported to exhibit excellent inter-rater reliability with an intraclass correlation coefficient of 0.95–0.99 and acceptable internal consistency as shown by a Cronbach's alpha of 0.78 (Williams et al., 2008). The excellent inter-rater reliability has been previously reported for the Japanese clinical population (Tabuse et al., 2007).

#### Secondary outcomes and other measures

Secondary outcomes included the severity of anxiety assessed by the Structured Interview Guide for Hamilton Anxiety Rating Scale (SIGH-A) (Shear et al., 2001), overall severity and improvement rated by the clinical global impression (CGI-S, CGI-I) (Guy, 1976), responder status defined by a ⩾50% reduction in the GRID-HAMD score, remission of symptoms defined as a GRID-HAMD score of <8 (Frank et al., 1991), and loss of a principal diagnoses as assessed by SCID-IV-TR at baseline. The reliability and validity for the SIGH-A have been previously demonstrated for the Japanese clinical population (Yamamoto, Aizawa, Inagaki, & Inada, 2012). These secondary measures (except for loss of diagnosis) were assessed at 10, 21, and 43 weeks. We cautiously assessed any adverse events using the Japanese version of the Common Terminology Criteria for Adverse Events (CTCAE v4.0) (US Department of Health & Human Services, 2009). The therapists or independent evaluators actively solicited patients at each visit to assess the occurrence or exacerbation of any adverse symptoms as well as their severity, duration, and relation to the study. A ‘serious’ adverse event included death, life-threatening, admission to the hospital, prolongation of hospitalization, disability, permanent damage, or a congenital anomaly/birth defect. The Credibility/Expectancy Questionnaire was administered at session 2 to assess the treatment expectancy (Devilly & Borkovec, 2000). The detailed psychometric properties of these measures for Western and Japanese populations have been described in a study protocol (Ito et al., 2016).

#### Sample size estimation

We initially set our minimum sample size to a total of 54 based on an assumed effect size of −0.85 for UP-TAU *v.* WL-TAU for the reduction of the primary outcome assessed by GRID-HAMD [see detailed discussion by Ito et al. (Ito et al. 2016)]. However, in the middle of the ongoing trial, the original developer laboratory of the UP at Boston University reported that the effect size of the UP against the wait-list condition on the HAMD at post-treatment was −0.69 (95% CI −1.06 to −0.31) in the definitive clinical trial of UP (Farchione et al., 2016). In response to this report, we re-estimated our sample size to detect a more conservative between-group effect size of −0.60 with a statistical power of 80% and a significance level of 0.05 with the two-tailed test. This resulted in a required sample size of 45 for each group. Considering the reported dropout rate of 15.39% (Farchione et al., 2012), we required at least 52 participants in each group to test the primary hypothesis of this study. This protocol amendment without any interim analysis was discussed with an ethicist in medicine, a biostatistician, and a psychiatrist with expertise in clinical research, all of whom belonged to the Department of Clinical Research Support at the NCNP, and was approved by the institutional review board (original approval number: A2013-092; approval number for the modified protocol: 2016-065), and timely changed in the records of ClinicalTrial.Gov (NCT02003261).

### Statistical analysis

All analyses for testing the efficacy with the primary and secondary measures were analyzed based on the intent-to-treat principle using a linear mixed model (LMM). For the primary outcome, the dependent variable was the GRID-HAMD score and the independent variables were assignment (i.e. UP-TAU *v.* WL-TAU), time (i.e. 0, 10, and 21 weeks), and interaction between the assignment and time as fixed-effect variables and participants as a random effect variable. We constructed a conditional growth model (Singer & Willett, 2003) using a restricted maximum likelihood estimation method to compare changes in depression severity between groups from baseline to 21 weeks. The assessment period comprised the measurement time for the growth model (i.e. 0, 10, and 21 weeks). We used unstructured error covariance for this model. To test robustness, we conducted the same LMM, including a stratified variable (depressive *v.* anxiety disorder) as a covariate, and other sensitivity analyses taking into account the difference between intention-to-treat and per-protocol analysis (Thabane et al., 2013). Continuous secondary outcomes (SIGH-A, CGI-S, and CGI-I) were analyzed in the same way as primary outcomes. The LMM were conducted using nlme package (Version: 3.1-142) in R version 3.6.2. Standardized effect size was calculated because it is preferable in comparison to simple effect size when comparing conceptually similar effects using different units of measurement (Baguley, 2009). Dichotomous secondary outcomes, such as responder status, remission status, and loss of principal diagnosis were analyzed using the incidence proportion difference and ratio.

Missing data were imputed with multiple imputations by chained equation using mice package (3.7.0) in R version 3.6.2 (van Buuren & Groothuis-Oudshoorn, 2011). The percentage of missing values for GRID-HAMD, SIGH-A, CGI-S, CGI-I, and loss of diagnosis were between 6.5% and 10.1%. All except one missing value was observed due to patient dropout. We did not observe any relationship between worsened or improved outcomes and missing data. Based on the assumption of missing at random, the results across 100 imputed datasets were combined by following Rubin's rule. We constructed multiple regression models, which included 15 demographic and clinical variables (e.g. gender, current occupation, principal diagnosis, months from first psychiatric appointment, and previous psychiatric hospitalizations). For all analyses, *p* < 0.05 was considered statistically significant. Maintenance of and/or continuing improvement were examined by calculating the within effect size from post-treatment (at 21 weeks) to follow-up (at 43 weeks). We calculated the occurrence of adverse events during the intervention and follow-up period. These analytic strategies were described in the published study protocol (Ito et al., 2016); however, the statistical analysis plan was not pre-registered in any open repository.

### Blinded data interpretation

We employed the blinded interpretation procedure to avoid interpretation bias for trial results (Järvinen et al., 2014). In brief, the epidemiologist who was blinded to randomization conducted statistical analysis for the primary and secondary outcomes. The results that used the labels group ‘U’ and group ‘P’ were provided to the principal investigator. The steering committee conducted a blinded interpretation meeting. The consensus was documented in June 2020. Then, an external reviewer, who had experience in randomized controlled trials for psychotherapy and was not involved in any aspect of this study, examined the documents. After this external validation, the data manager broke the randomization code. Records of the blinded data interpretation meeting are provided in the online Supplementary materials.

## Results

### Description of the participants

**Fig. 1.**
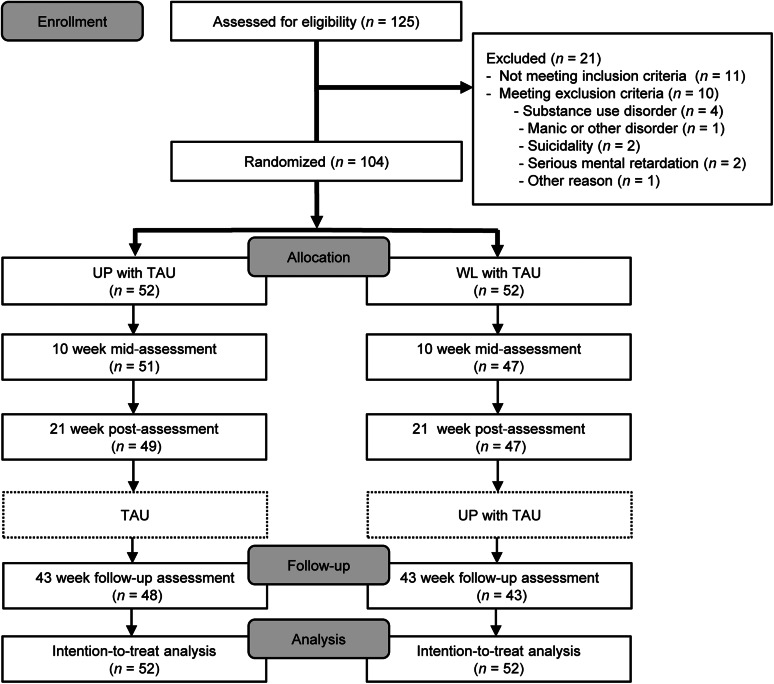
Participant flow diagram of the study.

Eligibility was assessed for 125 participants, of which 11 did not meet the inclusion criteria, and 10 met the exclusion criteria (Fig. 1). Table 1 shows the demographic and clinical characteristics at baseline. The mean age of the eligible participants was 37.4 (s.d. = 11.5). More than half of the participants (*n* = 62, 59.6%) met ⩾1 comorbid diagnoses. Furthermore, 26 out of the 54 participants (48.2%) with a principal diagnosis of depressive disorder had comorbidity with any anxiety disorder, whereas 26 out of the 50 participants (52.0%) with a principal diagnosis of anxiety disorder had comorbidity with any depressive disorder. The time range from the first psychiatric appointment was 2–498 months (mean = 93.8, s.d. = 93.0, first quartile = 16.75, median = 77, third quartile = 133.25).

**Table 1.** Baseline demographic and clinical characteristics

	Total (*n* = 104)	UP-TAU (*n* = 52)	WL-TAU (*n* = 52)
Age, mean (s.d.), y	37.4 (11.5)	37.02 (11.8)	37.79 (11.1)
Female	63 (60.6)	31 (59.6)	32 (61.5)
Marital status			
Single	55 (52.9)	27 (51.9)	28 (53.8)
Married	44 (42.3)	23 (44.2)	21 (40.4)
Divorced	5 (4.8)	2 (3.8)	3 (5.8)
Current occupation			
Employment	26 (25.0)	14 (26.9)	12 (23.1)
Education	8 (7.7)	4 (7.7)	4 (7.7)
Medical leave from job	13 (12.5)	6 (11.5)	7 (13.5)
Medical leave from education	4 (3.8)	3 (5.8)	1 (1.9)
Homemaker	15 (14.4)	6 (11.5)	9 (17.3)
Part-time job	10 (9.6)	6 (11.5)	4 (7.7)
Retired	28 (26.9)	13 (25.0)	15 (28.8)
Education level			
Elementary school	1 (1.0)	0 (0.0)	1 (1.9)
Junior high school	1 (1.0)	1 (1.9)	0 (0.0)
High school	27 (26.0)	14 (26.9)	13 (25.0)
Professional school	15 (14.4)	4 (7.7)	11 (21.2)
Two-year college	9 (8.7)	5 (9.6)	4 (7.7)
University/college	43 (41.3)	25 (48.1)	18 (34.6)
Graduate school	8 (7.7)	3 (5.8)	5 (9.6)
Principal diagnosis as stratified variable			
Depressive disorder	54 (51.9)	26 (50.0)	28 (53.8)
Anxiety disorder	50 (48.1)	26 (50.0)	24 (46.2)
Principal diagnosis			
Major depressive disorder	53 (51.0)	26 (50.0)	27 (51.9)
Dysthymia	1 (1.0)	0 (0.0)	1 (1.9)
Panic disorder with agoraphobia	13 (12.5)	5 (9.6)	8 (15.4)
Agoraphobia without history of panic disorder	5 (4.8)	2 (3.8)	3 (5.8)
Social anxiety disorder	14 (13.5)	7 (13.5)	7 (13.5)
Generalized anxiety disorder	4 (3.8)	3 (5.8)	1 (1.9)
Obsessive-compulsive disorder	9 (8.7)	5 (9.6)	4 (7.7)
Post-traumatic stress disorder	3 (2.9)	2 (3.8)	1 (1.9)
Anxiety disorder not otherwise specified	2 (1.9)	2 (3.8)	0 (0.0)
Number of comorbid diagnoses			
0	42 (40.4)	22 (42.3)	20 (38.5)
1	32 (30.8)	17 (32.7)	15 (28.8)
2	21 (20.2)	6 (11.5)	15 (28.8)
3	8 (7.7)	6 (11.5)	2 (3.8)
4	1 (1.0)	1 (1.9)	0 (0.0)
Comorbid diagnoses			
Depressive disorder	31 (29.8)	16 (30.8)	15 (28.8)
Anxiety disorder	71 (68.3)	35 (67.3)	36 (69.2)
Major depressive disorder	26 (25.0)	14 (26.9)	12 (23.1)
Dysthymia	5 (4.8)	2 (3.8)	3 (5.8)
Panic disorder with agoraphobia	9 (8.7)	3 (5.8)	6 (11.5)
Panic disorder without agoraphobia	1 (1.0)	1 (1.9)	0 (0.0)
Agoraphobia without history of panic disorder	10 (9.6)	5 (9.6)	5 (9.6)
Social phobia (social anxiety disorder)	15 (14.4)	7 (13.5)	8 (15.4)
Generalized anxiety disorder	8 (7.7)	4 (7.7)	4 (7.7)
Obsessive-compulsive disorder	17 (16.3)	8 (15.4)	9 (17.3)
Post-traumatic stress disorder	3 (2.9)	2 (3.8)	1 (1.9)
Anxiety disorder not otherwise specified	1 (1.0)	1 (1.9)	0 (0.0)
Other	7 (6.7)	4 (7.7)	3 (5.8)
Psychotropic medication			
SSRI	39 (37.5)	20 (38.5)	19 (36.5)
SNRI	13 (12.5)	7 (13.5)	6 (11.5)
Tricyclic antidepressant	7 (6.7)	4 (7.7)	3 (5.8)
NaSSA	11 (10.6)	8 (15.4)	3 (5.8)
Benzodiazepine	60 (57.7)	30 (57.7)	30 (57.7)
Antipsychotics	20 (19.2)	10 (19.2)	10 (19.2)
Months from first psychiatric appointment (mean, s.d.)	93.8 (93.0)	83.2 (88.5)	104.4 (96.2)
Number of previously utilized medical institutions (Mean, s.d.)	2.6 (2.0)	2.63 (1.8)	2.56 (2.1)
Previous psychiatric hospitalizations	19 (18.3)	12 (23.1)	7 (13.5)
Previous psychological counseling	50 (48.1)	26 (50.0)	24 (46.2)
Habitual use of alcohol	36 (35.6)	19 (36.5)	17 (32.7)
Habitual use of tobacco	21 (20.8)	15 (28.8)	6 (11.5)

Data are presented as the number (proportion) of patients unless otherwise indicated. UP-TAU, Unified Protocol with Treatment As Usual; WL-TAU, Wait-list with Treatment As Usual; SSRI, Selective Serotonin Reuptake Inhibitors; SNRI, Serotonin Norepinephrine Reuptake Inhibitors; NaSSA, Noradrenergic and Specific Serotonergic Antidepressant.

### Attrition, treatment expectancy, and treatment adherence

Of the 52 participants, 49 completed the UP intervention (94.2%), while 47 participants maintained the WL-TAU condition (90.4%). The mean number of completed sessions was 15.15 (s.d. = 3.10) for the UP-TAU group. The median treatment duration was 133.0 (first quartile = 125.8, third quartile = 140.0) days. The total number of sessions among the UP-TAU group was 788. Of these sessions, 309 assessed for adherence and 318 assessed for the fidelity; 217 sessions (87.4%) completely adhered to the treatment procedure. The mean treatment fidelity score was 4.01 (s.d. = 0.54). The mean total score of treatment expectancy in the UP-TAU group was 63.46 (s.d. = 18.19; possible range, 0–100).

### Assessment integrity and blinding success

To examine the inter-evaluator reliability of GRID-HAMD, 86 out of 406 (21.18%) randomly selected assessments were used. The single-measure inter-rater intraclass correlation coefficient (Model 2.1) between two evaluators was 0.96 (95% CI 0.94–0.98) for the total GRID-HAMD score. The intraclass correlation coefficient at the item level of GRID-HAMD (*n* = 1462) was 0.95 (95% CI 0.94–0.95).

The true treatment assignment was accurately assumed in 64 out of 98 cases at 10 weeks (65.3% correct, χ^2^ = 9.87, *p* < 0.01, 6 missing), 67 out of 96 cases at 21 weeks (69.8% correct, χ^2^ = 16.48, *p* < 0.01, 8 missing), and 58 out of 86 cases at 43 weeks (67.4% correct, χ^2^ = 13.70, *p* < 0.01, 18 missing). The number of ‘not at all sure’ responses of the assessors' certainty rating for their assumption was 69 out of 98 cases (70.4%) at 10 weeks, 71 out of 96 cases (74.0%) at 21 weeks, and 56 out of 86 cases (65.1%) at 43 weeks.

### Primary outcome

**Fig. 2.**
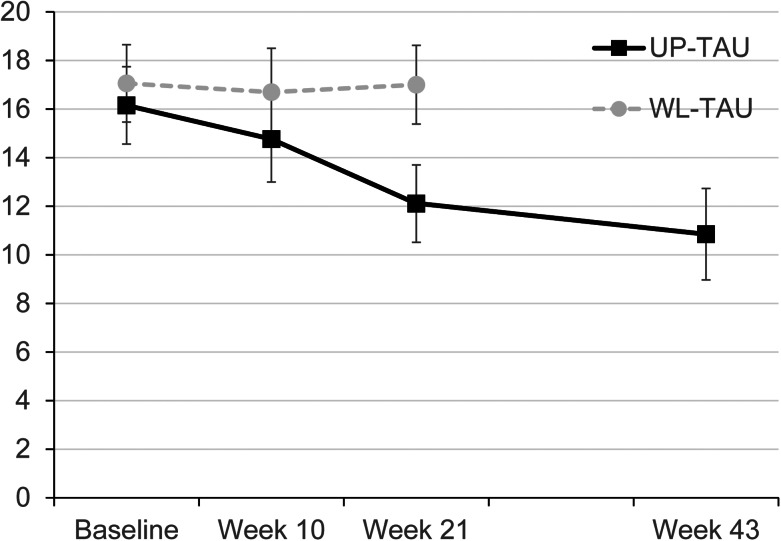
Course of patient improvement as measured with the GRID-Hamilton depression rating scale. Error bars represent 95% confidence intervals. UP, unified protocol; TAU, treatment-as-usual; WL, wait-list.

Table 2 shows the mean and standard deviation of GRID-HAMD at the baseline, 10-week mid-assessment, 21-week post-assessment, and 43-week follow-up. Figure 2 shows the improvement in the primary outcome measure. The mean GRID-HAMD scores in the UP-TAU and WL-TAU groups were 16.15 (s.d. = 4.90) and 17.06 (s.d. = 6.46) at baseline and 12.14 (s.d. = 5.47) and 17.34 (s.d. = 5.78) at 21 weeks. As shown in Table 3, the LMM analysis showed a significant difference in the GRID-HAMD score over the primary time point of 21 weeks between the UP-TAU and WL-TAU groups (estimate = −3.99; 95% CI −6.10 to −1.87). The estimated changes from baseline to 21 weeks were −4.06 (s.d. = 4.92) for the UP-TAU group and −0.32 (s.d. = 5.64) for the WL-TAU group. The between-group standardized mean difference was −0.70 (95% CI −1.11 to −0.28).

**Table 2.** Descriptive statistics of primary and secondary outcome measures by time point and treatment group

	0-week Baseline				10-week Mid-assessment				21-week Post-assessment		43-week Follow-up	
	UP-TAU	WL-TAU	UP-TAU	WL-TAU	UP-TAU	WL-TAU	UP-TAU	WL-TAU
Variable	*n* = 52		*n* = 52		*n* = 51		*n* = 47		*n* = 49	*n* = 47	*n* = 48	*n* = 43
GRID-HAMD	16.15 (4.90)		17.06 (6.46)		14.78 (5.99)		17.13 (6.71)		12.14 (5.47)	17.34 (5.78)	10.83 (6.62)	11.30 (6.53)
SIGH-A	21.12 (5.97)		21.69 (6.38)		20.20 (7.17)		22.00 (7.09)		17.82 (7.17)	22.11 (6.86)	15.10 (7.90)	16.58 (7.64)
CGI-S	4.39 (0.90)		4.33 (1.16)		4.12 (1.11)		4.40 (1.20)		3.43 (1.11)	4.28 (1.12)	2.94 (1.20)	2.88 (1.21)
CGI-I	−	−	−	−	3.69 (0.92)		3.92 (0.90)		2.98 (1.04)	3.79 (1.11)	2.56 (1.04)	2.35 (0.89)
Responder status	−	−	−	−	4 (7.69)		3 (5.77)		8 (15.39)	1 (1.92)	14 (26.92)	14 (26.92)
Remission status	−	−	−	−	7 (13.46)		2 (3.85)		10 (19.23)	3 (5.77)	18 (34.62)	16 (30.77)
Loss of principal Dx	−	−	−	−	−	−	−	−	1 (1.92)	0 (0.00)	6 (11.54)	8 (15.39)

Data are presented as mean (standard deviation) for GRID-HAMD, SIGH-A, CGI-A, and CGI-I and number (proportion) for responder status, remission status, and loss of principal diagnosis. Participants allocated to WL-TAU received UP during the follow-up period. UP-TAU, Unified Protocol with Treatment As Usual; WL-TAU, Wait-list with Treatment As Usual; GRID-HAMD, GRID-Hamilton Rating Scale for Depression; SIGH-A, Structured Interview Guide for Hamilton Rating Scale for Anxiety; CGI-S, Clinical Global Impression Severity; CGI-I, Clinical Global Impression Improvement; Dx, diagnosis.

**Table 3.** Primary and secondary outcomes

	UP-TAU		WL-TAU		WL-TAU compared with UP-TAU			Cohen's *d*		
						95%CI			95%CI	
Measure	Change from baseline to Post	(s.d.)	Change from baseline to Post	(s.d.)	Estimate	Lower	Upper		Lower	Upper
Primary outcome measure										
GRID-HAMD	−4.06	4.92	−0.32	5.64	−3.99	−6.10	−1.87	−0.70	−1.11	−0.28
Secondary outcome measures (continuous numeric variables)										
SIGH-A	−3.55	6.60	−0.17	6.77	−3.36	−6.05	−0.68	−0.50	−0.90	−0.09
CGI-S	−0.98	1.04	−0.11	0.91	−0.87	−1.27	−0.48	−0.88	−1.30	−0.46
CGI-I	−	−	−	−	−0.80	−1.45	−0.16	−0.74	−1.15	−0.32
Secondary outcome measures (dichotomous variables)	Incidence proportion				Incidence proportion difference	95%CI		Incidence proportion ratio	95%CI	
						Lower	Upper		Lower	Upper
Responder status	16.80		2.45		14.35	1.82	26.87	7.27	0.89	59.67
Remission status	20.69		7.01		13.68	−1.35	28.71	3.02	0.83	10.95
Loss of primary diagnosis	2.29		0.35		1.94	−37.31	41.18	1.02	0.69	1.51

UP-TAU, Unified Protocol with Treatment As Usual; WL-TAU, Wait-list with Treatment As Usual; GRID-HAMD, GRID-Hamilton Rating Scale for Depression; SIGH-A, Structured Interview Guide for Hamilton Rating Scale for Anxiety; CGI-S, Clinical Global Impression Severity; CGI-I, Clinical Global Impression Improvement.

### Secondary outcomes

The descriptive statistics for secondary outcomes are shown in Table 2. As shown in Table 3, the LMM analysis showed significant differences in the changes of SIGH-A (estimate = −3.36; 95% CI −6.05 to −0.68), CGI-S (estimate = −0.87; 95% CI −1.27 to −0.48). The estimated change of SIGH-A and CGI-S from baseline to 21 weeks was −3.55 (s.d. = 6.60) and −0.98 (s.d. = 1.04) in the UP-TAU group, respectively. The LMM analysis showed a significant difference in CGI-I at 21 weeks (estimate = −0.80; 95% CI −1.45 to −0.16). The mean CGI-I was 2.98 (s.d. = 1.04) for the UP-TAU group at 21 weeks. The Cohen's *d* values (95% CI) for SIGH-A, CGI-S, and CGI-I were −0.50 (−0.90 to −0.09), −0.88 (−1.30 to −0.46), and −0.74 (−1.15 to −0.32), respectively.

For the dichotomous outcomes, eight out of 52 (15.4%) patients in the UP-TAU group and one out of 52 (1.9%) patients in the WL-TAU group exhibited a treatment response at 21 weeks, resulting in an incidence proportion ratio of 7.27 (95% CI 0.89–59.67). The remission status was met in 10 patients (19.2%) in the UP-TAU group and three patients (5.8%) in the WL-TAU group, resulting in an incidence proportion ratio of 3.02 (95% CI 0.83–10.95). A loss of the principal diagnosis was observed in one patient in the UP-TAU group and in no patients in the WL-TAU group, resulting in an incidence proportion ratio of 1.02 (95% CI 0.69–1.51).

### Sensitivity analyses for controlling stratified variables and using per-protocol samples

The LMM analyses that included stratified variables (i.e. the principal diagnosis of depressive *v.* anxiety disorders) as covariates showed significant differences between the UP-TAU and WL-TAU groups regarding the changes in GRID-HAMD, SIGH-A, CGI-S, and CGI-I over the primary time point of 21 weeks (see online Supplementary materials, Table S1). The LMM analyses using per-protocol samples revealed consistent results, as significant differences were observed between the UP-TAU and WL-TAU groups regarding the changes in GRID-HAMD, SIGH-A, CGI-S, and CGI-I (see online Supplementary materials, Table S2).

### Maintenance of treatment effect during the follow-up period

Descriptive statistics at follow-up are shown in Table 2. The post- to follow-up effect sizes of each outcome for the UP-TAU group were not significant for GRID-HAMD, SIGH-A, and CGI-I (see online Supplementary materials, Table S3). The significant within effect size in CGI-S showed further improvement in patients in the UP-TAU group (Hedges' *g* = −0.48; 95% CI −0.95 to −0.01).

### Adverse events

Table 4 shows the occurrence of adverse events. No serious adverse events were observed for the UP-TAU or WL-TAU groups during the intervention period. However, eight out of 52 participants (15.4%) reported a total of 13 intervention-related adverse events during the intervention period of which 10 events were rated as mild, as defined by CTCAE (missing data = 3). The mean duration of adverse events was 24.15 (s.d. = 16.72; range: 5–70) days.

**Table 4.** Summary of adverse events

	Intervention period				Follow-up period			
	UP-TAU (*n* = 52)		WL-TAU (*n* = 52)		UP-TAU (*n* = 48)		WL-TAU (*n* = 43)	
	Total	Related to study	Total	Related to study	Total	Related to study	Total	Related to study
Number of patients reporting AEs	29	8	11	0	8	0	22	7
Number of AEs reported	64	13	28	0	12	0	56	13
Number of SAEs reported	0	0	0	0	2	0	2	0
AEs by type								
Psychological	25	7	6	0	4	0	25	6
Medical	39	6	22	0	10	0	33	7
Psychological AEs								
Anxiety	12	4	1	0	2	0	11	4
Depression	8	1	3	0	0	0	4	0
Suicidal ideation	0	0	0	0	1	0	1	0
Irritability/Anger	2	1	0	0	0	0	8	2
Other	3	1	2	0	1	0	1	0
Medical AEs								
Sleepiness	4	0	1	0	0	0	2	0
Malaise	5	1	1	0	2	0	4	0
Insomnia	5	2	5	0	1	0	0	0
Throat discomfort	3	1	0	0	0	0	0	0
Dizziness	1	1	2	0	0	0	1	0
Gastrointestinal problems	2	0	1	0	0	0	1	1
Low-back pain	1	0	0	0	0	0	2	0
Nausea	3	0	1	0	0	0	1	1
Headache	2	0	0	0	1	0	7	2
Cold symptoms	2	0	1	0	1	0	0	0
Other	11	1	10	0	5	0	15	3

Participants allocated to WL-TAU received UP during the follow-up period. UP-TAU, Unified Protocol with Treatment As Usual; WL-TAU, Wait-list with Treatment As Usual; AE, Adverse Event; SAE, Serious Adverse Event.

## Discussion

This is the first randomized controlled trial to test the efficacy of the UP that includes either a principal diagnosis of depressive or anxiety disorder. As hypothesized, the addition of the UP to TAU was more effective than waiting for the UP while receiving TAU with regards to improving the severity of depressive symptoms between baseline and post-treatment assessments. The UP-TAU group showed significant improvements compared with the WL-TAU group in the blinded assessor evaluation of anxiety, severity, and improvement of clinical global impression. In the UP-TAU group, improvements from the baseline to 21 weeks were maintained in all continuous outcomes at 43 weeks. The sensitivity analyses controlling the types of principal diagnosis and per-protocol sample analyses showed the robustness of these results. There was no significant difference regarding the treatment response, the remission status, and the loss of primary diagnosis at 21 weeks between the UP-TAU and WL-TAU groups. The proportion of dropout was low in the UP-TAU group (3/52, 5.8%). No serious adverse events and few non-serious adverse events occurred in the UP-TAU group during the intervention period.

The UP was effective for improving primary and most of the secondary outcomes. Compared to the largest clinical trial of the UP, our participants seemed to exhibit more severe depressive and anxiety symptoms (our trial: mean baseline GRID-HAMD = 16.15, s.d. = 4.90, mean SIGH-A = 21.12, s.d. = 5.97, Barlow et al., 2017: mean HAMD = 11.55, s.d. = 7.02, mean SIGH-A = 17.06, s.d. = 8.50). Moreover, half of our participants exhibited comorbidity of both depressive and anxiety disorders. Our between-group effect sizes comparing wait-list at post-treatment (−0.70 for HAMD and −0.50 for SIGH-A) were consistent with Barlow's trial (−0.57 for HAMD and −0.53 for SIGH-A). Overall, our results extend the evidence regarding the efficacy of the UP not only to patients with a principal diagnosis of PD, SAD, GAD, and OCD but also to patients with a principal diagnosis of depressive disorders and with more severe depressive and anxiety symptoms. Our efficacy results were consistent with those of prior RCTs with similar designs (Bameshgi et al., 2019; Marnoch, 2014). In addition, our results show that UP is effective for participants who have a prolonged history of receiving usual treatments. For example, approximately 6 years (median = 77 months) had passed since our participants had their first psychiatric appointment. Most of our participants had visited at least two other medical institutions before visiting our treatment site, and approximately 20% of them had a history of psychiatric hospitalizations [*n* = 19, 23.1% (UP-TAU), 13.5% (WL-TAU)].

Despite the efficacy evidence, our results suggest that the UP-TAU is not sufficient for most of our patients to fully recover from clinical conditions. The mean reduction in GRID-HAMD was 4.01, which is within the range of 1 s.d. at baseline and post-treatment. The mean CGI-S for the UP-TAU group at 43 weeks was 2.94, indicating that the patients were still ‘mildly ill.’ A CGI-S score of 3 means that the patient shows mild symptoms with minimal distress or difficulty in social and/or occupational function. Only one at 21 weeks and eight at 43 weeks out of 54 participants achieved the loss of principal diagnosis. Our participants in the UP-TAU group showed only 15.4% treatment response (i.e. ⩾50% GRID-HAMD score reduction) and 19.2% remission (i.e. a GRID-HAMD score of <8) at 21 weeks. Though these were improved at 43 weeks (26.9% and 34.6%, respectively), these were still lesser compared with the treatment response proportion of 41% (95% CI 38–43) and the remission (i.e. a GRID-HAMD score of <7) proportion of 26% (95% CI 20–33) in various psychotherapies (Cuijpers et al., 2021). However, our results of poor response proportion might partly be attributable to the participant characteristics in this study, because the proportion of response in WL condition was also poor compared with the results of meta-analysis (Our participant 5.5% *v.* mean of 92 trials 16%) (Cuijpers et al., 2021). Despite the relatively long treatment periods, these dichotomous outcomes were insignificant. Participants who showed a partial response to UP may require additional treatment for residual symptoms. Although our results support the efficacy of UP for depressive and anxiety disorders, there is room for improvement in the content or administration of UP to individual patients.

Our results provide additional evidence regarding the feasibility of the UP with TAU in a Japanese clinical setting. No serious and only a few (*n* = 13) non-serious study-related adverse events occurred for eight out of 52 patients in the UP-TAU group (15.4%) during the intervention period. This is within the range of expected undesired events of 5%–20% for psychotherapy patients (Linden & Schermuly-Haupt, 2014). The treatment fidelity score was good, which is consistent with Barlow's trial (4.44 *v.* 4.01 in our study). The rate of dropout in our study (5.8%) was low compared to previous trials (Barlow et al., 2017; 12.5%, Marnoch, 2014; 12.5%, Bameshgi et al., 2019; 11.8%). The mean expectancy scale score was consistent with the trial conducted by treatment developers (Sauer-Zavala et al., 2018) [63.46 (our study) *v.* 65.71 (Barlow's study)]. The major approach for psychiatric treatment for depressive and anxiety disorders in Japan is prescribing psychotropic medications with very brief routine outpatient examinations. Unfortunately, the provision of CBT for depression did not increase in the 6 years after it was first covered by Japan's national insurance scheme in 2010 (Hayashi et al., 2020). Several factors are thought to have contributed to the current situation. These include an insufficient number of trained CBT therapists. Transdiagnostic psychotherapies, such as UP, are expected to reduce the cost and burden of training practitioners in multiple evidence-based treatments (Martin et al., 2018; McHugh & Barlow, 2010; Murray et al., 2019). Our results offer valuable insights on the possible applications of UP in global mental health, particularly its extended applicability to depressive disorders and the Asian population.

This study must be interpreted while considering the following limitations. First, although we intentionally included heterogeneous clinical populations to test the efficacy of UP for depressive and anxiety disorders, such broad inclusion of participants might hinder the interpretation of the results in comparison with previous clinical trials conducted under traditional diagnostic systems. Second, the statistical test did not indicate blinding success. The proportion of the evaluators' correct guesses was 69.8% at the primary time point. This is relatively higher than that of randomized controlled trials regarding schizophrenia and affective disorders (62.0%) (Baethge, Assall, & Baldessarini, 2013), though 74% (71/96) of the assessment was conducted under the conditions in which the evaluator's guess was ‘not at all sure.’ Third, our comparison group consisted of the condition of waiting for the UP while receiving TAU. We selected this condition because it is the most clinically relevant comparison in our Japanese setting. However, the nocebo effect might have affected our results. Fourth, although our participants were clinically heterogeneous, they were homogenous regarding race, ethnicity, and nationality. We must be cautious regarding the generalizability across national or cultural backgrounds. Fifth, there are no long-term follow-up data. Sixth, this study was not powered to test multiple outcomes. Therefore, our results for secondary outcomes and our sensitivity analyses should be interpreted as explorative. Seventh, we only reported the results regarding the pre-determined primary and secondary outcomes; all these outcomes were assessed by blinded assessors.

## Conclusion

This study provides evidence that the addition of UP on TAU can be efficacious for patients with depressive and/or anxiety disorders in reducing depression. Specifically, our results strengthen the evidence of UP for patients with a principal diagnosis of MDD. Because our results regarding treatment response, remission, and loss of diagnosis were weak, future studies need to investigate strategies to enhance these aspects, for example, by examining moderating and mediating factors.

## Supplementary material

For supplementary material accompanying this paper visit https://doi.org/10.1017/S0033291721005067. click here to view supplementary material
