# Supplementary material for: Efficacy of the unified protocol for transdiagnostic cognitive-behavioral treatment for depressive and anxiety disorders: a randomized controlled trial
Source: Psychol Med. 2022 Jan 10;53(7):3009–20. doi: 10.1017/S0033291721005067 (PMC10235654; doi:10.1017/S0033291721005067)
Supplement: Supplementary file 1 [file S0033291721005067sup.zip › S0033291721005067sup002.pdf]

Blinded Data Analyses Statement of Interpretation

Tokyo, Japan, July 1, 2020.

Title of the trial:

Japan Unified Protocol Clinical Trial for Depressive and Anxiety Disorders (JUNP Study)

Clinical Trial Registry:

NCT02003261

UMIN000030708

The Steering Committees of the Blinded Interpretation of the JUNP Study:

Masaya Ito, Masaru Horikoshi, Noriko Kato, Yuki Oe, Hiroko Fujisato, Keiko Yamaguchi, Shun Nakajima, Mitsuhiro Miyamae, Ayaka Toyota, and Yoshitake Takebayashi

Purpose of this document:

The Steering Committees of the JUNP study (listed above) is aimed at developing and recording two interpretations of the results based on a blinded review of the primary outcome data (treatment U compared to treatment P), with one assuming that U was the intervention group (unified protocol plus treatment-as-usual) and another assuming that U was the comparison group (waiting-list with treatment-as-usual).

Background assumptions (theoretical basis/commitments and previous knowledge for data analysis and scientific goal of being objective and free of preconceptions):

The unified protocol for the transdiagnostic treatment of emotional disorders is a promising treatment approach. It could be applicable to a broad range of mental disorders, including depressive, anxiety, trauma-related, and obsessive-compulsive disorders. However, no randomized controlled trial has been conducted to verify the efficacy of the individual format of the unified protocol on the heterogeneous clinical population with depressive and anxiety disorders.

The trial was designed as a single-center, assessor-blinded, randomized, 20-week, parallel-group superiority study to compare the efficacy of the combination of the unified protocol and treatment-as-usual (UP-TAU) versus waiting-list with treatment-as-usual (WL-TAU) for patients with depressive and/or anxiety disorders. The primary outcome was depression at 21 weeks, assessed by the 17-item version of the GRID-Hamilton Rating Scale for Depression.

Statistical Commitment:

All analyses for testing the efficacy of treatment on primary and secondary measures will be analyzed based on the intent-to-treat principle, using a linear mixed model (LMM).

For the primary outcome, the dependent variable is the GRID-HAMD score, and the independent variables are assignment (i.e., intervention vs. control group), time (i.e., 0, 10, and 21 weeks), and interaction of the assignment and time, as a fixed-effect variable, and participants, as a random-effects variable.

Secondary outcomes will be analyzed in the same way as the primary outcomes.

Based on these theoretical commitments, our interpretation of the findings will be as follows:

a) If UP-TAU is found superior to WL-TAU, the study is applicable to the patient group studied, but its generalizability remains unknown.

b) If UP-TAU is not found superior to WL-TAU, the study suggests that UP-TAU does not work at all.

Summary of key statistical analyses

Table 2. Descriptive statistics of primary and secondary outcome measures by time point and treatment group

	0 week Baseline				10 week Mid assessment				21 week Post assessment				43 week Follow-up			
	U group		P group		U group		P group		U group		P group		U group		P group	
Variable	*n* = 52		*n* = 52		*n* = 51		*n* = 47		*n* = 49		*n* = 47		*n* = 48		*n* = 43	
GRID-HAMD (Mean, SD)	16.15	4.90	17.06	6.46	14.78	5.99	17.13	6.71	12.14	5.47	17.34	5.78	10.83	6.62	11.30	6.53
SIGH-A(Mean, SD)	21.12	5.97	21.69	6.38	20.20	7.17	22.00	7.09	17.82	7.17	22.11	6.86	15.10	7.90	16.58	7.64
CGI-S(Mean, SD)	4.39	0.90	4.33	1.16	4.12	1.11	4.40	1.20	3.43	1.11	4.28	1.12	2.94	1.20	2.88	1.21
CGI-I(Mean, SD)	–	–	–	–	3.69	0.92	3.92	0.90	2.98	1.04	3.79	1.11	2.56	1.04	2.35	0.89
Responder status (Number, proportion)	–	–	–	–	4	7.7	3	5.8	8	15.4	1	1.9	14	26.9	14	26.9
Remission status (Number, proportion)	–	–	–	–	7	13.5	2	3.8	10	19.2	3	5.8	18	34.6	16	30.8
Loss of principal diagnosis (Number, proportion)	–	–	–	–	–	–	–	–	1	1.9	0	0.0	6	11.5	8	15.4

Table 3. Primary and Secondary outcomes

	U group		P group		P group compared with U group					
						95%CI		Cohen's *d*	95%CI	
Measure	Change from baseline to Post	(*SD*)	Change from baseline to Post	(*SD*)	Estimate	Lower	Upper		Lower	Upper
Primary outcome measure										
GRID-HAMD	-4.06	4.92	-0.32	5.64	-3.99	-6.10	-1.87	-0.70	-1.11	-0.28
Secondary outcome measures (continuous numeric variables)										
SIGH-A	-3.55	6.60	-0.17	6.77	-3.36	-6.05	-0.68	-0.50	-0.90	-0.09
CGI-S	-0.98	1.04	-0.11	0.91	-0.87	-1.27	-0.48	-0.88	-1.30	-0.46
CGI-I	–	–	–	–	-0.80	-1.45	-0.16	-0.74	-1.15	-0.32
Secondary outcome measures (dichotonomous variables)	Incidence proportion				Incidence proportion difference	95%CI Lower	95%CI Upper	Incidence proportion ratio	95%CI Lower	95%CI Upper
Responder status	18.33		6.48		11.85	-2.85	26.54	3.30	0.53	20.61
Remission status	22.46		11.17		11.29	-5.61	28.19	2.13	0.63	7.16
Loss of primary diagnosis	7.58		9.33		-1.75	-13.20	9.70	0.81	0.21	3.20

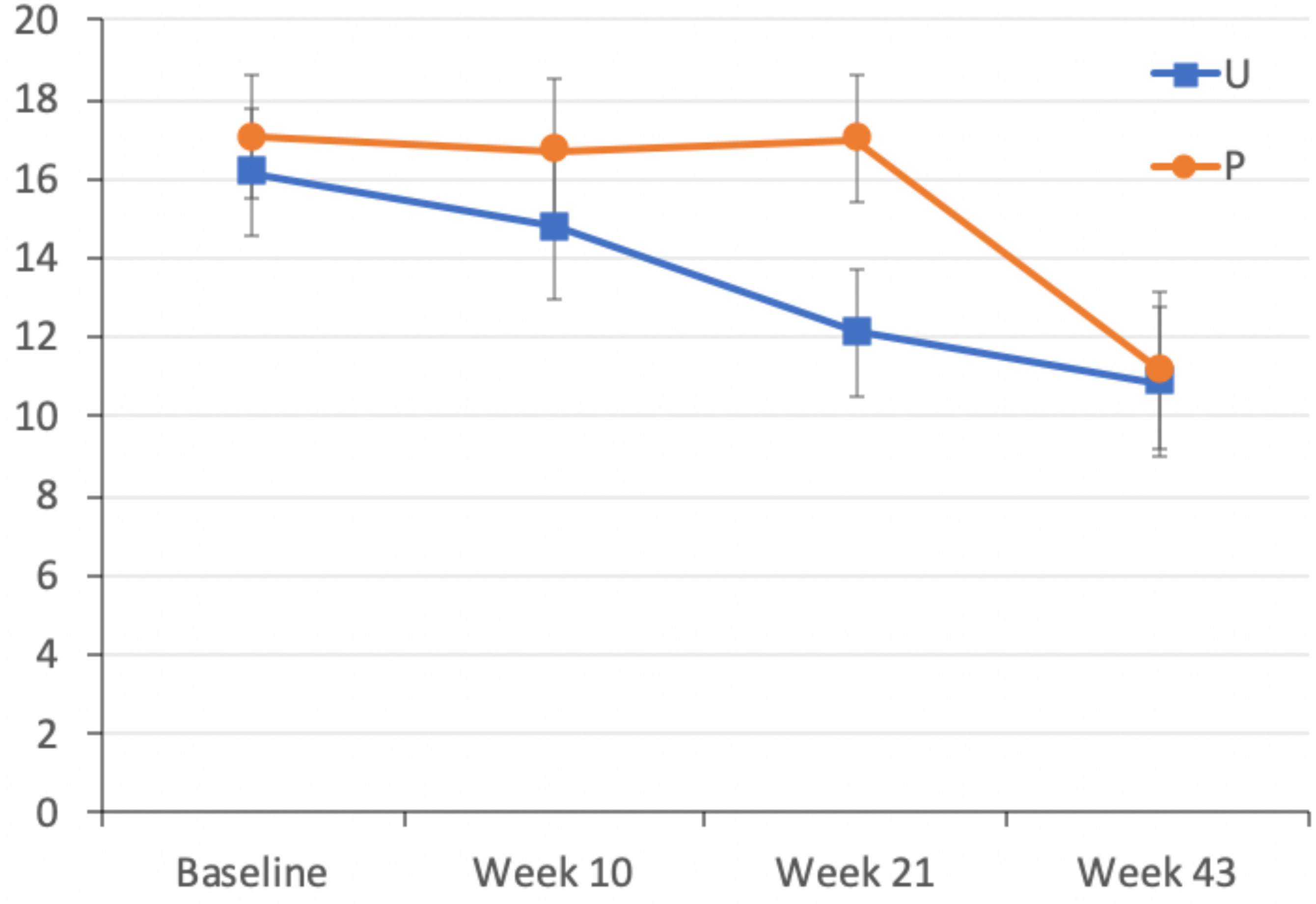

Fig 2. Course of improvement on the GRID-Hamilton depression rating scale.

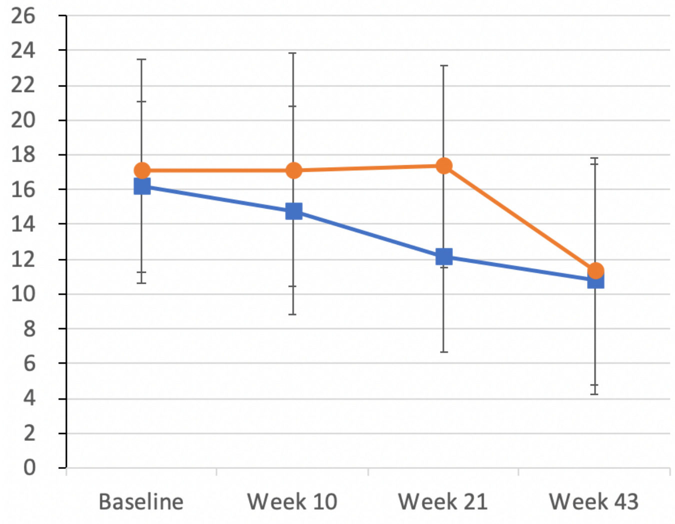

Fig B1. Course of improvement on the GRID-HAMD (Mean, SD)

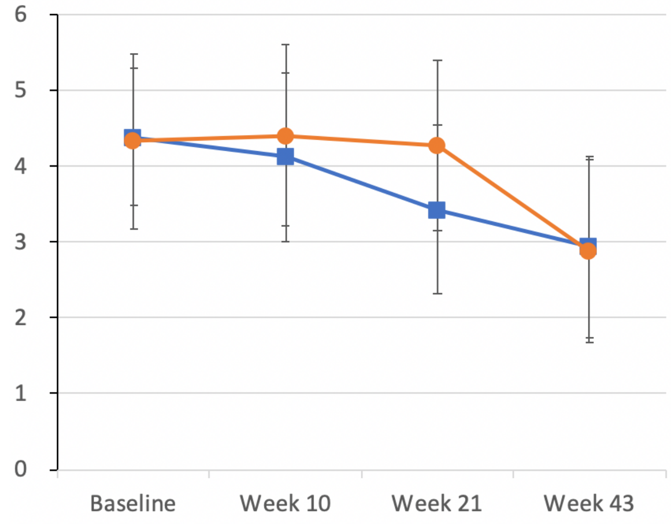

Fig B3. Course of improvement on the CGI -S (Mean, SD)

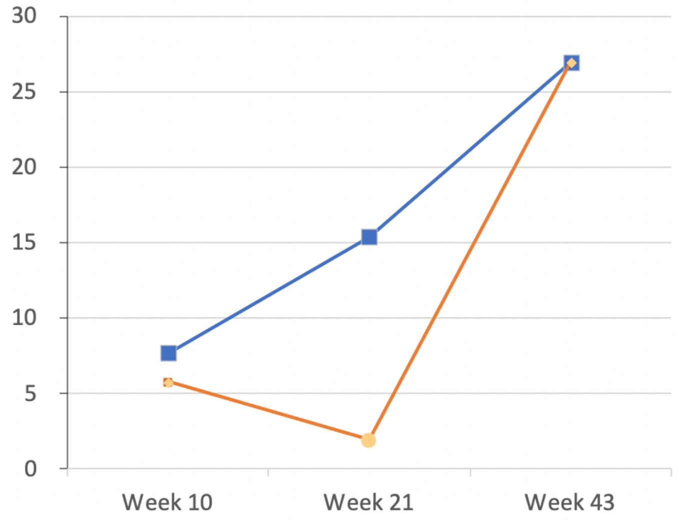

Fig B5. Proporton of Responder status

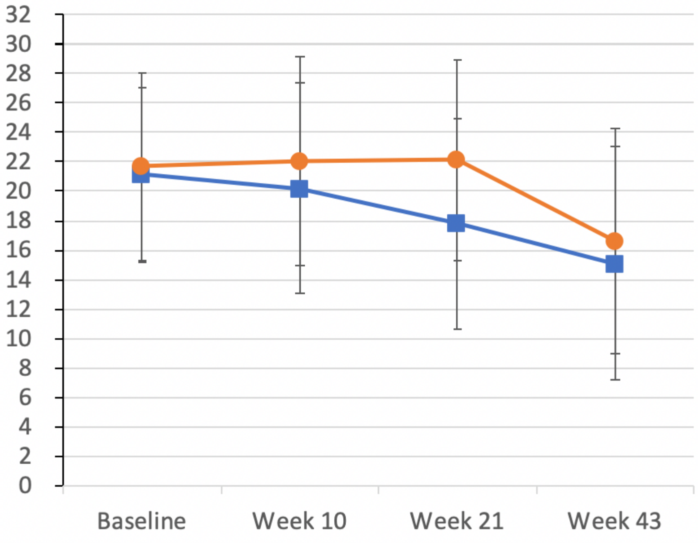

Fig B2. Course of improvement on the SIGH-A (Mean, SD)

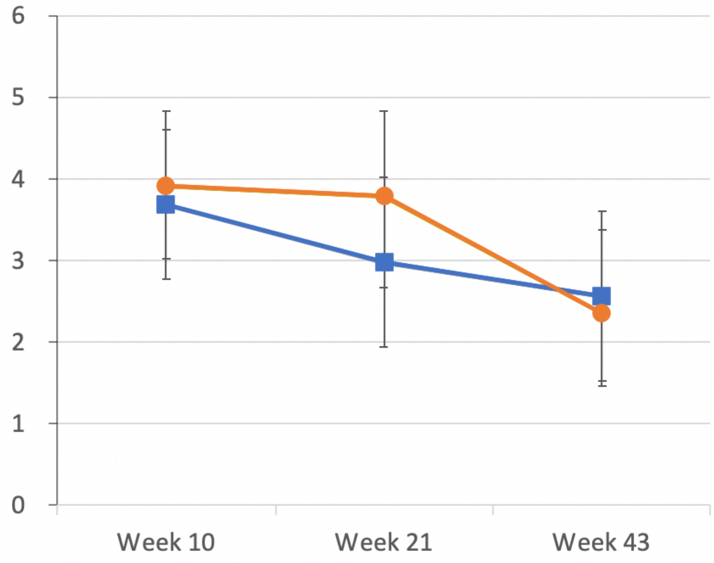

Fig B4. Course of improvement on the CGI-I (Mean, SD)

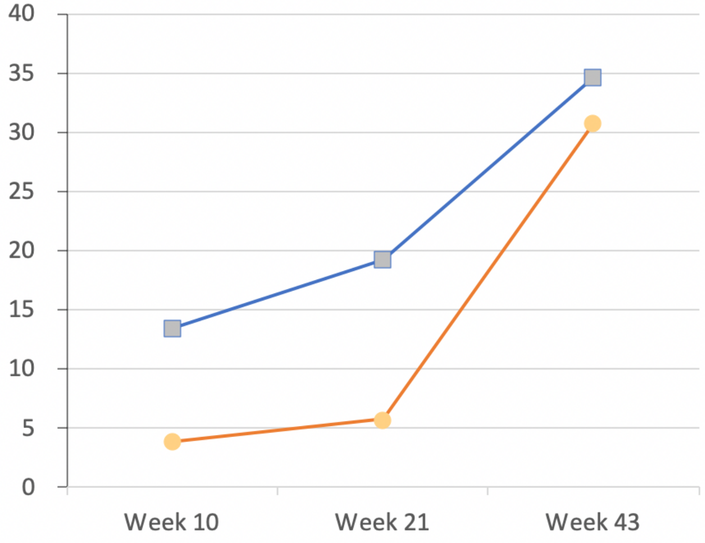

Fig B6. Proporton of Remission status

Minutes of the blinded review of the data

1) **If Group U = UP-TAU and Group P = WL-TAU**

Group U showed a decrease in the score of GRID-HAMD over time, whereas group P showed no change in the score until week 21 and then a decrease in week 43. The LMM analysis showed a significant difference between the groups at the primary time point (week 21). If group U is the intervention group (UP-TAU), the results support our primary hypothesis. Namely, UP is effective for treating depressive symptoms among patients with a principal diagnosis of depressive or anxiety disorders. The magnitude of the decrease is slightly smaller than that in our pilot trial (Ito et al., 2016; Pilot trial score Baseline M = 22.1, SD = 5.5, Post M = 11.9, SD = 4.9; this study baseline M = 16.15, SD = 4.90, Post = 12.14, SD = 5.47). This difference could be attributable to the change in eligibility criteria between the pilot and definitive study. Because the inclusion criteria of GRID-HAMD somatization factor ≥ 7 in the pilot trial was changed to the total score of GRID-HAMD ≥ 8 in our definitive trial, the participants of this study were patients with less severe symptoms. Alternatively, the lower magnitude of change might be attributable to a more rigorous design of the trial, including central randomization and masked assessment by independent evaluators in the definitive trial (Cuijpers et al., 2016; Cuijpers et al., 2020). The maintenance of symptoms in group P (WL-TAU) might reflect the Nocebo effect of the wailing list period (Furukawa et al., 2014). Compared to the largest trial of UP with anxiety and obsessive-compulsive disorders, our participants seemed to have more severe depressive and anxiety symptoms (Our trial: Baseline GRID-HAMD M = 16.15, SD = 4.90, SIGH-A M = 21.12 SD = 5.97; Barlow et al. 2017, HAMD M = 11.55, SD = 7.02, SIGH-A M = 17.06, SD = 8.50). Because the effect sizes for these measures were consistent between the studies, our results extend the efficacy evidence of UP not only to patients with a principal diagnosis of anxiety- and obsessive-compulsive disorders but also to patients with a principal diagnosis of depressive disorders and with more severe depressive and anxiety symptoms.

The efficacy of UP is strengthened by significant differences in secondary outcomes (anxiety, and clinical global impressions) at the primary time point (week 21). Along with a low proportion of dropouts in group U at week 21 (49/52, 5.8%), these efficacy results showed that UP is an effective and acceptable intervention as the concurrent treatment with usual care in the Japanese setting.

2) **If Group U = WL-TAU and Group P = UP-TAU**

Group U showed a decrease in the score of GRID-HAMD over time, whereas group P showed no change in the score until week 21 and then a decrease to week 43. The LMM analysis showed a significant difference between the groups at the primary time point (week 21). If group U is the control group (WL-TAU), the results does not support our primary hypothesis. It could be interpretable that the decrease in group U reflects the effect of "treatment-as-usual." Moreover, it could be interpreted that the non-improvement in group P (UP-TAU) reflects the harmful effect of UP for patients with depressive and anxiety disorders. In other words, UP has prevented or minimized the treatment effect of treatment-as-usual. This interpretation could also be supported with the decrease in

symptoms from week 21 to 43 in group P (UP-TAU), that is, during the period of the participant relief from UP intervention. The preventive effect might be attributable to the emotional exposure of UP in that patients are continuingly asked to accept and tolerate their negative emotions. These treatment procedures might be making patients keep experiencing negative emotional symptoms, as assessed by GRID-HAMD and SIGH-A. Other possibility might be that the treatment effect of group P (UP-TAU) appeared in a delayed manner. However, this interpretation should be abandoned because group U (WL-TAU) showed continued improvement until week 43, showing the enduring treatment effect of TAU. This means that TAU is sufficient for participants' symptom improvement, and there is no additional treatment effect of UP.

Barlow DH, Farchione TJ, Bullis JR, et al. The Unified Protocol for Transdiagnostic Treatment of Emotional Disorders Compared With Diagnosis-Specific Protocols for Anxiety Disorders: A Randomized Clinical Trial. JAMA Psychiatry. 2017;74(9):875–884. doi:10.1001/jamapsychiatry.2017.2164

Cuijpers, P., Cristea, I. A., Karyotaki, E., Reijnders, M., & Huibers, M. J. (2016). How effective are cognitive behavior therapies for major depression and anxiety disorders? A meta-analytic update of the evidence. World psychiatry : official journal of the World Psychiatric Association (WPA), 15(3), 245–258. https://doi.org/10.1002/wps.20346

Cuijpers P, Karyotaki E, de Wit L, Ebert DD. The effects of fifteen evidence-supported therapies for adult depression: A meta-analytic review. Psychother Res. 2020;30(3):279-293. doi:10.1080/10503307.2019.1649732

Furukawa TA, Noma H, Caldwell DM, et al. Waiting list may be a nocebo condition in psychotherapy trials: a contribution from network meta-analysis. Acta Psychiatr Scand. 2014;130(3):181–192. doi:10.1111/acps.12275

Ito M, Horikoshi M, Kato N, et al. Transdiagnostic and Transcultural: Pilot Study of Unified Protocol for Depressive and Anxiety Disorders in Japan. Behav Ther. 2016;47(3):416–430. doi:10.1016/j.beth.2016.02.005
