# Supplementary figures and images for: Efficacy of the unified protocol for transdiagnostic cognitive-behavioral treatment for depressive and anxiety disorders: a randomized controlled trial
Source: Psychol Med. 2022 Jan 10;53(7):3009–20. doi: 10.1017/S0033291721005067 (PMC10235654; doi:10.1017/S0033291721005067)

## Slide 1
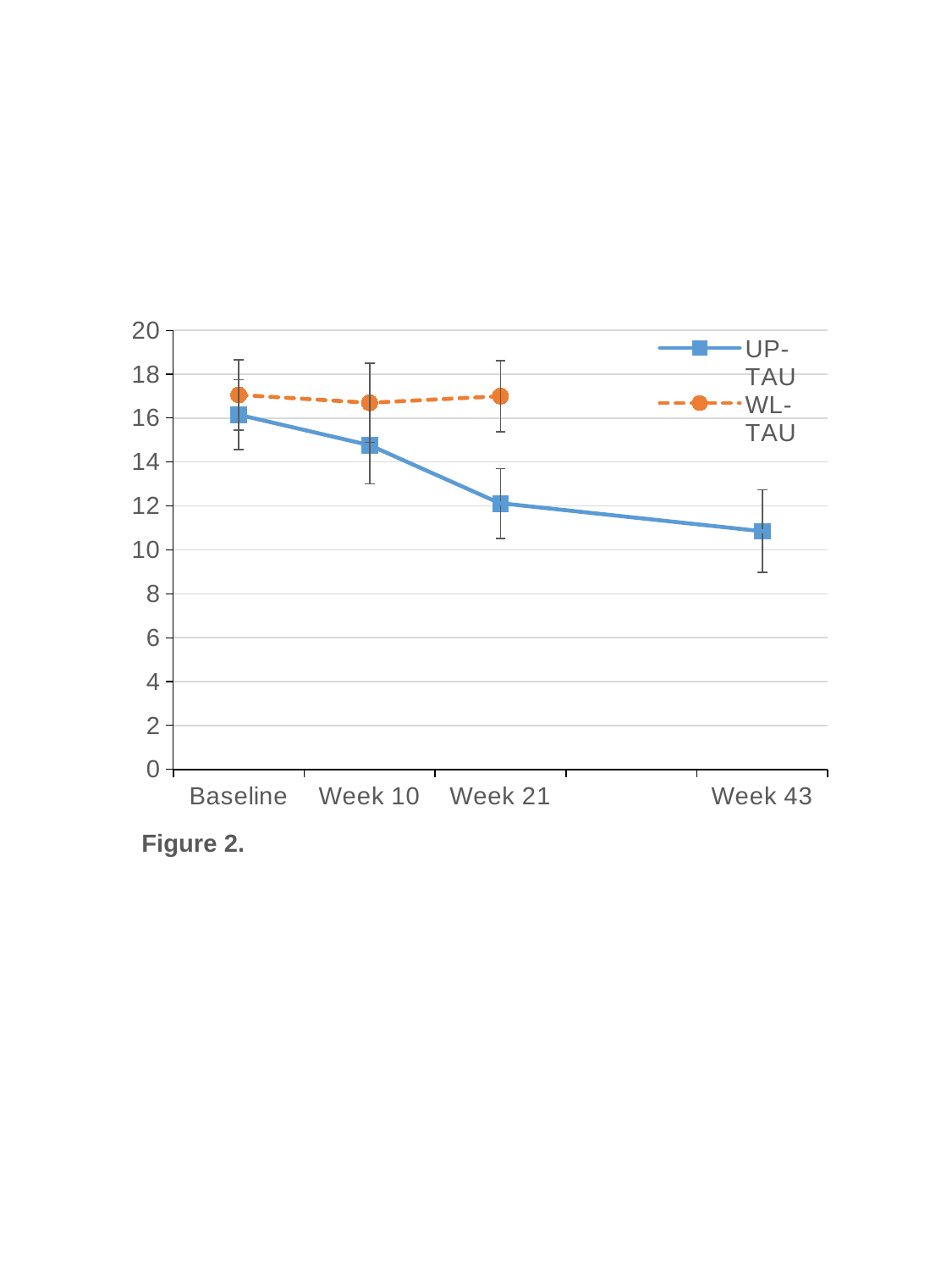

### Chart
| Category | UP-TAU | WL-TAU |
|---|---|---|
| Baseline | 16.1538461538461 | 17.0576923076923 |
| Week 10 | 14.7668300302583 | 16.6990727457278 |
| Week 21 | 12.1100210666269 | 17.0027015338644 |
| | None | None |
| Week 43 | 10.8510307074723 | None |Figure 2.

Supplement: Supplementary file 1 [file S0033291721005067sup.zip › S0033291721005067sup003.pptx]
